# Use of a Smartphone Application for Spine Surgery Improves Patient Adherence with Preoperative Instructions and Decreases Last-minute Surgery Cancellations

**DOI:** 10.7759/cureus.4192

**Published:** 2019-03-06

**Authors:** Jeffrey J Stewart, Islam Fayed, Shawnda Henault, Babak Kalantar, Jean-Marc Voyadzis

**Affiliations:** 1 Miscellaneous, Georgetown University School of Medicine, Washington DC, USA; 2 Neurosurgery, Medstar Georgetown University Hospital, Washington DC, USA; 3 Orthopaedics, Medstar Georgetown University Hospital, Washington DC, USA

**Keywords:** mobile applications, patient satisfaction, perioperative care, smartphone, surgical cancellations

## Abstract

Background: Smartphone applications (apps) are being increasingly utilized in the health care arena to improve patient care and outcomes.

Objective: To further demonstrate the ability of a smartphone app to improve patient compliance with preoperative instructions and to decrease the number of last-minute surgery cancellations.

Methods: Patients undergoing spine surgery were prospectively accrued. Smartphone app users were compared to non-app users. Patient adherence with preoperative instructions as well as last-minute surgery cancellations were analyzed.

Results: All 85 app users adhered to preoperative instructions according to the acknowledgements sent to the web portal, and there were no cancelled surgeries. Among the 89 non-app users, there were five cancelled surgeries (5.6%).

Conclusions: We demonstrate the ability of a smartphone application to improve patient adherence with preoperative instructions and decrease last-minute surgery cancellations.

## Introduction

Smartphone applications (apps) are being increasingly utilized to elevate the quality of patient care. In the surgical setting, improving patient adherence with preoperative instructions can improve outcomes and patient satisfaction while reducing last-minute surgical cancellations, which represent a substantial loss of revenue to hospitals and surgeons. While some studies have already yielded preliminary results, this is an area in need of further investigation to establish the role of smartphone app in effectively addressing these patient-centered metrics [1].

We previously reported our initial experience with a smartphone app in the perioperative care of neurosurgery patients with promising findings [2]. A prospective comparative analysis is presented to evaluate the ability of such an app to improve patient adherence with preoperative instructions and reduce last-minute surgery cancellations in neurosurgery and orthopedics.

## Materials and methods

Patients who were able to download the Amie by FavorHealth app (Favorhealth Inc., Sarasota, Florida, USA) and had access to an Apple or Android smartphone or tablet were selected prospectively. Patients who did not satisfy these criteria were considered non-app users and were also followed. Non-app users were given standard paper instructions for surgery. Patient demographics were recorded. All patients underwent spinal surgeries in an outpatient or inpatient setting within the neurosurgery or orthopedic surgery departments at Medstar Georgetown University Hospital. Institutional Review Board (IRB) approval was not required due to the observational nature of the study.

**Figure 1:**
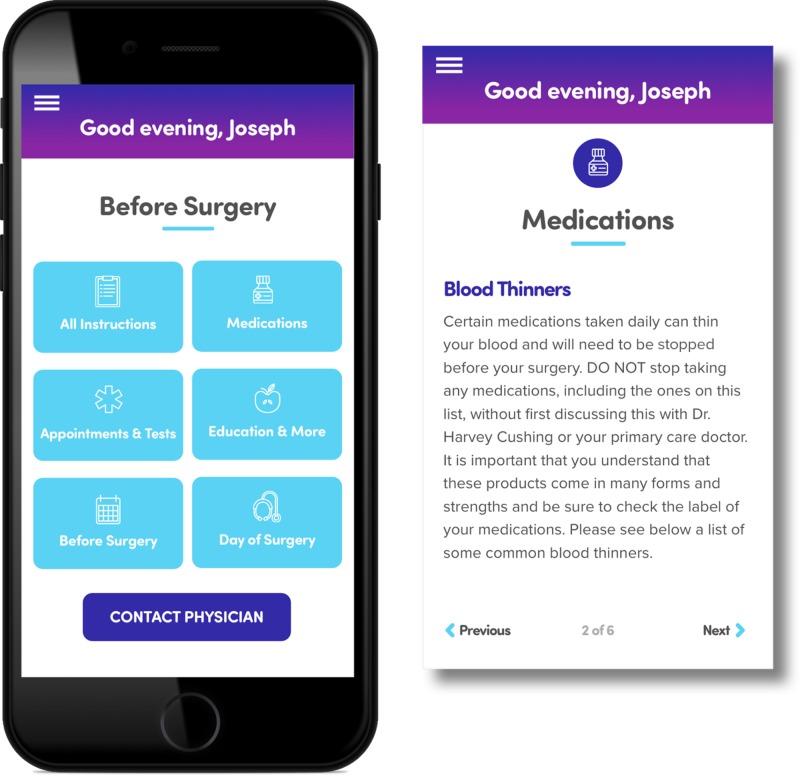
Patient Application Portal

Faculty and staff created specific pre- and postoperative instructions based on standard surgical templates. Surgery-specific instructions were tailored according to physician preference and were associated with reminders in the form of push notifications that patients would receive both before and after surgery. Reminders were focused on the most important elements that could lead to surgery cancellations: preoperative clearance evaluation by specialists, laboratory work, medication management (stopping non-steroidal anti-inflammatory medications or blood thinners), preoperative illness, and day-of-surgery instructions (Figure 1). To improve understanding of the proposed surgical intervention, hyperlinks from the patient’s app to websites and approved videos were added when appropriate.

Through the app, patients were prompted to acknowledge certain instructions and all reminders. These acknowledgements were sent back to the web portal preoperative dashboard in order for the physician’s staff to track compliance with instructions before surgery.

All parties (physicians, residents, physician assistants, nurse practitioners, office assistants, and patients) accepted the terms of use. The web portal and app were developed using a standard Health Insurance Portability and Accountability Act (HIPAA)-compliant privacy policy.

The primary end points were: 1) Compliance with instructions before surgery 2) Any preoperative surgery cancellation within 48 hours due to non-compliance with instructions (e.g., failure to maintain nil per os (NPO) status, obtain specialty clearance, or discontinue blood thinners). The group of patients using the app was compared to non-app users that did not meet the criteria for app use.

## Results

Patients undergoing routine elective spine procedures were prospectively accrued. There were 82 males and 94 females with an average age of 56 ± 14.0 (range 25 to 95) (Table 1).

**Table 1:** Patient Demographics

	App Users (n=85)	Non-App Users (n=89)
Average age ± SD	53 ± 13.2	59.7 ± 14.0
Sex		
Male	42	40
Female	43	49

Eighty-seven patients meeting inclusion criteria for app use agreed to participate in the study. Eighty-five of these 87 patients successfully downloaded and used the app. All 85 of these patients read and complied with preoperative instructions as determined by patient acknowledgements sent from the app to the physicians’ office through the app’s web portal (Table 2).

**Table 2:** Primary Endpoints

	App Users (n=87)	Non-App Users (n=89)
Downloaded and used the Amie app	85	N/A
Adherence with preoperative instructions	85	N/A
Last-minute Surgery Cancelations	0	5

There were no canceled surgeries in the group of app users compared to five canceled surgeries in the non-app user group (Table 2). The reasons for cancellations are presented in Table 3. Patients #1 and #2 were canceled on the day of surgery because of uncontrolled hypertension due to failure to comply with antihypertensive medications. Patients #3 and #4 had significant cardiac comorbidities and did not follow instructions to see their cardiologists prior to surgery. Patient #5 had advanced renal insufficiency and did follow instructions to see a nephrologist prior to surgery.

**Table 3:** Reasons for Surgery Cancellations Among Non-app Users

	Reason for Last-Minute Surgery Cancelations Among Non-App Users
Patient #1	Uncontrolled hypertension
Patient #2	Uncontrolled hypertension
Patient #3	Failure to obtain cardiology clearance
Patient #4	Failure to obtain cardiology clearance
Patient #5	Failure to obtain nephrology clearance

## Discussion

Last-minute surgery cancellations represent a substantial loss of revenue for hospitals, ambulatory surgery centers, and physicians. Elective surgery cancellation rates vary significantly in the literature, with some studies reporting ranges of 6% to 20% [3-9]. The estimated loss of revenue to hospitals ranges from $1,730 to $4,550 per surgical case or $1,430 to $1,700 per operating room hour according to several studies [3-5]. Cancellations compounded yearly and across disciplines costs hospitals millions of dollars. These data do not include the loss of revenue to the surgeon.

Various factors contribute to last-minute surgery cancellations, with many being patient-related and preventable. Specifically, Fayed et al. found that the four most common causes of same-day surgery cancellation for elective procedures were patient no-show (27%), need for further medical optimization (24.1%), operating room unavailability (19.3%), and patient refusal or lack of consent (8.8%) [10]. With the exception of operating room availability, the remaining three causes of surgery cancellations may be related to patient education and compliance with preoperative instructions, and thus represent potential areas for improvement to be targeted through the use of an app.

Patient retention of information presented in the clinic is limited [2]. While factors including the reduced time of clinic visits, as well as patient age and anxiety, may contribute to this, patients’ delayed recollection of information is still limited even when very thorough education is provided and the patients can correctly recall the information at the time of the visit [2]. Furthermore, large, generic packets of preoperative instructions place the burden of finding the relevant information at the appropriate time on the patient. Insufficient patient understanding and retention of the information presented at a preoperative visit can lead to poor patient adherence with instructions such as continuing antihypertensive medications, discontinuing blood thinners, or obtaining additional medical evaluation and clearance for surgery, and this, in turn, may lead to last-minute surgery cancellations due to the need for further medical optimization of the patient on the day of surgery [2].

A smartphone application may be employed to address these issues. Categorized tabs within the app allow the patient to easily find any of information discussed in the clinic that they wish to revisit, whether that be their diagnosis, the proposed procedure, tests they need to obtain, or changes they need to make to their medications. Furthermore, in-app reminders sent to the patient in the form of pop-up notifications act as a fail-safe to ensure that the patients remember to not only follow these instructions, but also to do so on the correct days [2]. Finally, the patient sends their acknowledgement of these instructions back to the provider to both keep them abreast of their progress and provide an opportunity for the provider to intervene if necessary, prior to the day of their surgery. The app may also effectively address patient no-shows with pop-up reminders leading up to the date of surgery [2]. Finally, Fayed et al. cited patient concerns and the need for further counseling as causes for failure of patients to provide consent for surgery [10]. An app that provides patients with easy to understand and readily accessible information about their upcoming procedure can both educate patients as well as alleviate some of their concerns prior to surgery [2].

In the present study, among the app users, there was 100% compliance with preoperative instructions as demonstrated by acknowledgements sent by patients through the app to the web portal. There were no last-minute surgery cancellations in the patient cohort of 85 app users. There were five surgery cancellations in the non-app users cohort yielding a cancellation rate of 5.6%. Reasons for cancellations were uncontrolled hypertension due to non-adherence with medications and failure to obtain cardiology and nephrology clearances.

Limitations of this study include a relatively small patient cohort and lack of randomization. Moreover, patients with smartphones or tablets who are facile with apps are generally younger and more likely to retain information when compared to older patients [2]. The median age of the app users was 53, whereas the median age of non-app users was 60. Patients with a more advanced age are more likely to be less compliant with instructions, thus possibly confounding the results of the study.

## Conclusions

The use of a perioperative care app with built-in reminders and a preoperative patient tracking dashboard improved compliance with instructions, while allowing physicians and their staff to track the patient’s journey before surgery. This led to a significant difference in last-minute surgery cancellations in a prospective comparative analysis of app and non-app users. A larger multi-institutional randomized study is needed to validate these findings and conclusively demonstrate clinical efficacy and cost savings.
